# Comprehensive risk score of the E-PASS scoring system serves a prognostic indicator for patients after neoadjuvant therapy and curative esophageal cancer surgery: a multicenter retrospective study

**DOI:** 10.3389/fonc.2025.1617683

**Published:** 2025-06-06

**Authors:** Nanjing Li, Yixin Liu, Jianfeng Zhou, Xiang Li, Shenglu Lian, Yushang Yang

**Affiliations:** ^1^ Division of Radiotherapy, Cancer Center, West China Hospital of Sichuan University, Chengdu, Sichuan, China; ^2^ Department of Thoracic Surgery, West China Hospital of Sichuan University, Chengdu, Sichuan, China

**Keywords:** esophageal cancer, curative resection, E-PASS, neoadjuvant, survival & prognosis

## Abstract

**Background:**

Neoadjuvant chemoradiotherapy (nCRT) and curative surgery have been recommended as the standard treatments for locally advanced esophageal cancer. Nevertheless, the postoperative morbidity and long-term survival outcomes for patients following this consensus treatment plan remain suboptimal. Therefore, preoperative risk assessment is essential to identify high-risk patients and predict adverse postoperative outcomes. This multicenter study aimed to evaluate the Estimation of Physiologic Ability and Surgical Stress (E-PASS) scoring system for predicting the short- and long-term outcomes of esophageal cancer patients treated with nCRT and curative esophagectomy.

**Methods:**

Patients with esophageal cancer who underwent curative resection between 2010 and 2022 were retrospectively enrolled in this study. The cohort was divided into the low and high comprehensive risk score (CRS) groups. The CRS cutoff value was determined using the Youden index applied to overall survival (OS) curves. Prognostic value was assessed through Cox regression and Kaplan–Meier analyses.

**Results:**

In total, 814 patients were enrolled, including 556 and 258 patients with low and high CRS, respectively. ROC curve analysis determined that the CRS was a highly specific and sensitive predictive tool for postoperative complication occurrence and severity (AUC=0.889 and 0.838, respectively). When the cutoff value was established using the Youden index applied to overall OS curves, multivariate analysis demonstrated that the CRS was an independent prognostic factor for OS (HR: 1.48; 95% CI 1.14–1.92, P=0.003) and recurrence-free survival (RFS) (HR: 1.44; 95% CI 1.13– 1.82, P=0.002). Furthermore, the Kaplan–Meier survival curves of OS and RFS also demonstrated high CRS group had worse long-term outcomes, irrespective of tumor regression scores and esophageal cancer stage.

**Conclusions:**

The E-PASS scoring system emerges as a visible predictor of short- and long-term outcomes in patients with esophageal cancer undergoing nCRT and curative surgery.

## Introduction

Esophageal cancer ranks the seventh most common type of cancer and the sixth leading cause of cancer-associated death worldwide, with over half of cases reported in East Asia (1, 2). Increasing evidence suggests that neoadjuvant chemoradiotherapy (nCRT) with surgery benefits the long-term outcomes of locally advanced esophageal cancer patients (3, 4). However, despite curative resection combined with nCRT has gradually become the standard treatment, over 40% patients still experience recurrence with poor prognosis (5, 6). In addition, the recently reported overall postoperative complication rates after esophagectomy for esophageal cancer are 11.6% to 35.6% in East Asia and 35.9% to 63.2% in the Western countries (7–11). Therefore, the comprehensive optimization of treatment strategies for locally advanced esophageal cancer patients is crucial. Ideally, to enhance preoperative risk assessment and shared decision-making, an esophageal cancer-specific risk model is essential, helping clinicians identify high-risk patients in a targeted manner. However, to date, there is no such model specifically tailored for esophageal cancer patients.

Patient-related factors and surgical variables are critical contributors to surgical risks, exerting influences on postoperative complication rates and impacting long-term prognosis (12). Two decades ago, Japanese researchers established a scoring system for predicting outcomes following elective gastrointestinal surgery (13, 14). This model hypothesized that postoperative complications result from a disruption of homeostasis caused by excessive surgical stress exceeding the patient’s reserve capacity. Subsequently, the model integrates preoperative and surgical variables to formulate the Estimation of Physiologic Ability and Surgical Stress (E-PASS), which comprises surgical stress score (SSS), preoperative risk score (PRS), and comprehensive risk score (CRS). Recently, the E-PASS model has demonstrated efficacy in predicting morbidity and mortality following various gastrointestinal surgeries, particularly validated within Asian populations (15, 16). However, the relationship between E-PASS scores and longterm clinical outcomes in esophageal cancer patients remains unclear, and the significance of this scoring system has not been fully elucidated (17). Moreover, there is a lack of research evaluating the applicability of the E-PASS model in a large sample of esophageal cancer patients across multiple institutions, especially those underwent nCRT combined with esophagectomy.

Currently, the long-term prognosis of esophageal cancer patients who underwent nCRT and esophagectomy is primarily predicted through pathological staging and tumor regression scores (TRS). Our hypothesis suggests that, in addition to evaluating pathological results, a comprehensive assessment of clinical and surgical factors through CRS is indispensable. This study aimed to analyze a multicenter dataset to evaluate the value of the E-PASS scoring system in predicting both short- and longterm outcomes in esophageal cancer patients with nCRT and esophagectomy.

## Method

### Participants

This multicenter retrospective study involved four high-volume institutions in China, including West China Hospital, Sanya People’s Hospital, Shangjin Nanfu Hospital and West China Tianfu Hospital. The retrospective screening process included patients who underwent curative esophagectomy at these institutions from July 2010 to January 2022. Meanwhile, the exclusion criteria included (1) Patients with unresectable cancer involving adjacent structures (T4) or distant metastasis (M1); (2) those with concomitant malignancies; (3) transhiatal procedure; (4) palliative resection. The Ethics Committee of West China Hospital of Sichuan University (No. 2022767) provided approval for this study, and the need for patient consent was waived.

### Oncological and surgical management

Regimens for nCRT included paclitaxel plus 5-fluorouracil plus cisplatin or cisplatin, combined with 45 Gy of concomitant radiotherapy. A preoperative assessment was recommended to evaluate surgical feasibility following 2–4 cycles of neoadjuvant regimens.

For resectability evaluation, all patients underwent baseline clinical examination, routine blood analyses, contrast-enhanced computed tomography (CT), electrocardiogram, and respiratory function testing. Additional diagnostic procedures, such as external ultrasound of the cervical region and positron emission tomography/computed tomography (PET/CT), were selectively performed. The standard surgical approach encompassed minimally invasive esophagectomy, open thoracotomy and hybrid procedure. When patients converted from minimally invasive to open surgery, the final surgical approach was recorded as open surgery. Patients without evidence of cervical lymph node metastasis on preoperative CT and ultrasound underwent routine two-field lymph node dissection, otherwise three-field lymph node dissection is carried out.

### E-PASS models

The E-PASS scoring system is essentially a regression model, as comprehensively described by Haga et al. (15). Briefly, a CRS was determined by combining a PRS with 6 clinical variables and a SSS comprising 3 surgical variables. The formulas for calculating these scores were as follows:

PRS = −0.0686 + 0.00345X1 + 0.323X2 + 0.205X3 + 0.153X4 + 0.148X5 + 0.0666X6, wherein X1 indicated age, X2 represented the presence (1 point) or absence (0 points) of severe heart disease (New York Heart Association class III-IV or severe arrhythmia requiring mechanical support), X3 denoted the presence (1 point) or absence (0 points) of severe pulmonary disease (vital capacity <60% or forced expiratory volume in 1 second <50%), X4 signified the presence (1 point) or absence (0 points) of type 1 or 2 diabetes (World Health Organization (WHO) criteria), X5 represented the WHO performance status index (range, 0–4 points), and X6 stood for the American Society of Anesthesiologists physiological status classification (range, 1–5 points).

SSS = −0.342 + 0.0139X1 + 0.0392X2 + 0.352X3, wherein X1 represented blood loss (in gram) normalized by body weight (in kilogram), X2 corresponded to the duration of the surgical procedure (in hour), and X3 denotes the scale of the skin incision (rated as 0 points for a minimally invasive esophagectomy, 1 point for thoracotomy alone, and 2 points for a hybrid of thoracotomy and minimally invasive esophagectomy).

CRS = −0.328 + 0.936 (PRS) + 0.976 (SSS). We computed the PRS utilizing data documented upon admission, followed by the inclusion of SSS data. The CRS was then calculated after esophagectomy. To further evaluate this effect within the same CRS strata, patients were classified into two subgroups: those with dominant physical burden (defined as PRS above the 75th percentile and SSS below the 25th percentile) and those with dominant operative burden (defined as SSS above the 75th percentile and PRS below the 25th percentile).

### Tumor pathology and regression evaluation

Tumor pathological characteristics, nodal status, and tumor regression were reviewed independently by two pathologists. Tumor regression was assessed based on the College of American Pathologists (CAP) criteria, including TRS0 (absence of viable tumor cells), TRS1 (presence of single cells or rare small groups of tumor cells), TRS2 (residual tumor with evident regression but more than single cells or rare small groups), and TRS3 (extensive residual tumor with no evident regression). Pathologic staging followed the 8th edition TNM staging system from the American Joint Committee on Cancer.

### Endpoints and follow-up

The primary endpoints included assessing the effectiveness of the CRS in predicting Overall Survival (OS) and Recurrence-Free Survival (RFS). Secondary endpoints focused on postoperative morbidity, with all complications within 30 days after surgery or during the hospital stay scored according to the Clavien-Dindo (CD) grading system (ranging from I to V) (18). Severe complication was specifically categorized as ClavienDindo grade II or higher. RFS was defined as the time period from the surgery date to the first documented relapse, or death from any cause. Patients who were alive or lost to follow-up were censored at their last follow-up date. During the initial year after surgery, patients were observed every 3 months, followed by 6 months thereafter. The last general follow-up of survivors was performed in January 2024.

### Statistical analysis

R programming language (version 4.3.2) and SPSS 25.0 software (IBM) were used for the data analysis. Normally distributed continuous variables underwent analysis through Student’s t-test, while non-normally distributed continuous variables were assessed using the Mann-Whitney U test. Categorical variables underwent comparison using the Chi-squared test or Fisher’s exact test as appropriate. For postoperative complication prediction, the cutoff value for CRS was derived from relevant receiver operating characteristic (ROC) curve analysis. The optimal cutoff point was established employing the Youden index (J = sensitivity + specificity - 1). In further investigation of patients demographic and oncological characteristics as well as long-term outcome, the cutoff value for high and low CRS groups was determined through the application of the Youden index to OS curves. The clinical utility of the ROC curves were evaluated using decision curve analysis (DCA) and clinical impact curve (CIC) via R software. Survival rates were estimated using the Kaplan–Meier method, and overall differences between survival curves were compared using the Cox proportional hazards model. A multivariate analysis was performed using a Cox proportional hazards model after significant prognostic variables were defined through a univariate analysis. Statistical significance was set at a two-sided P value of <0.05.

## Results

### E− PASS and patients characteristics

**Figure 1:**
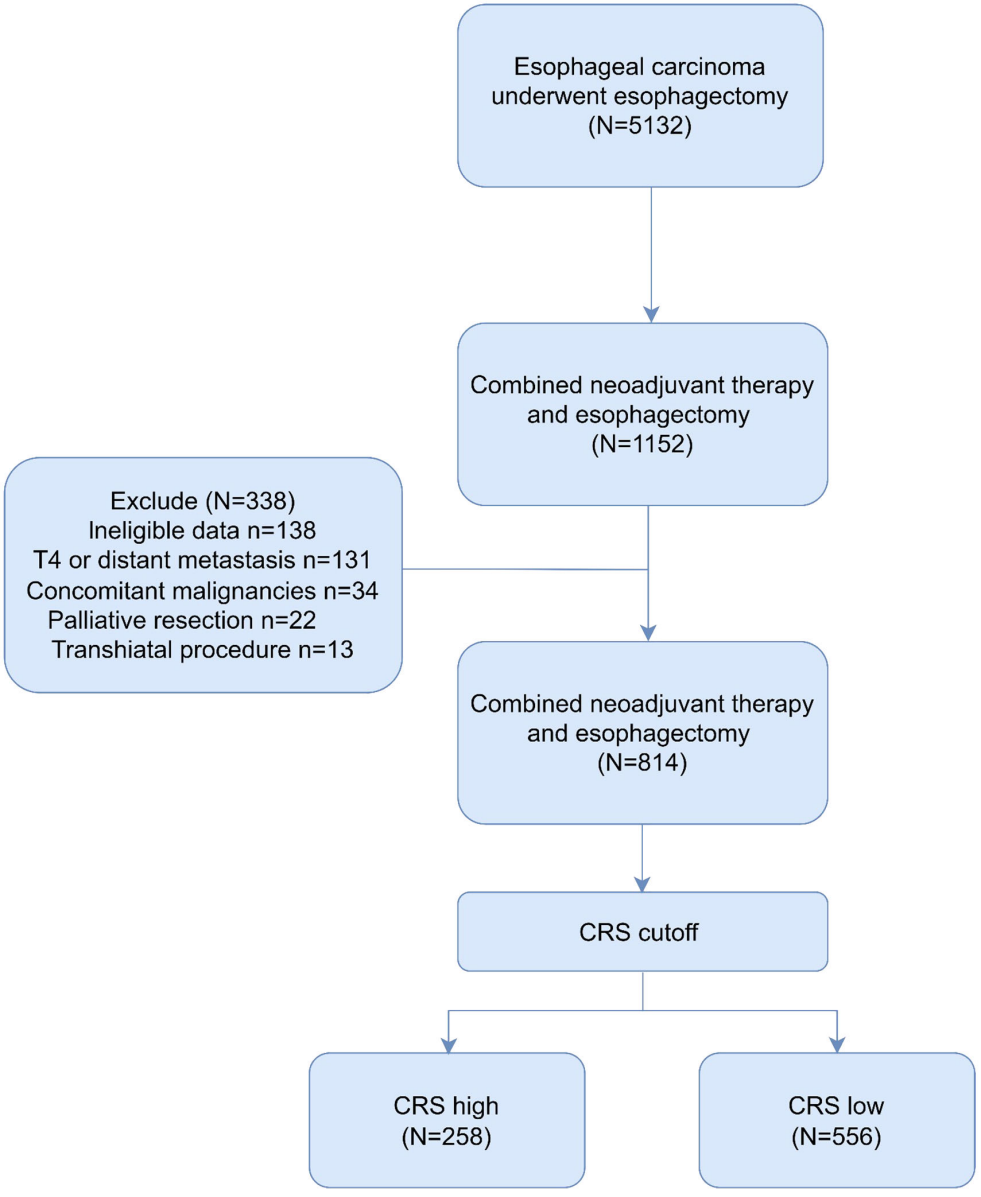
Flow chart of patient selection.

**Figure 2:**
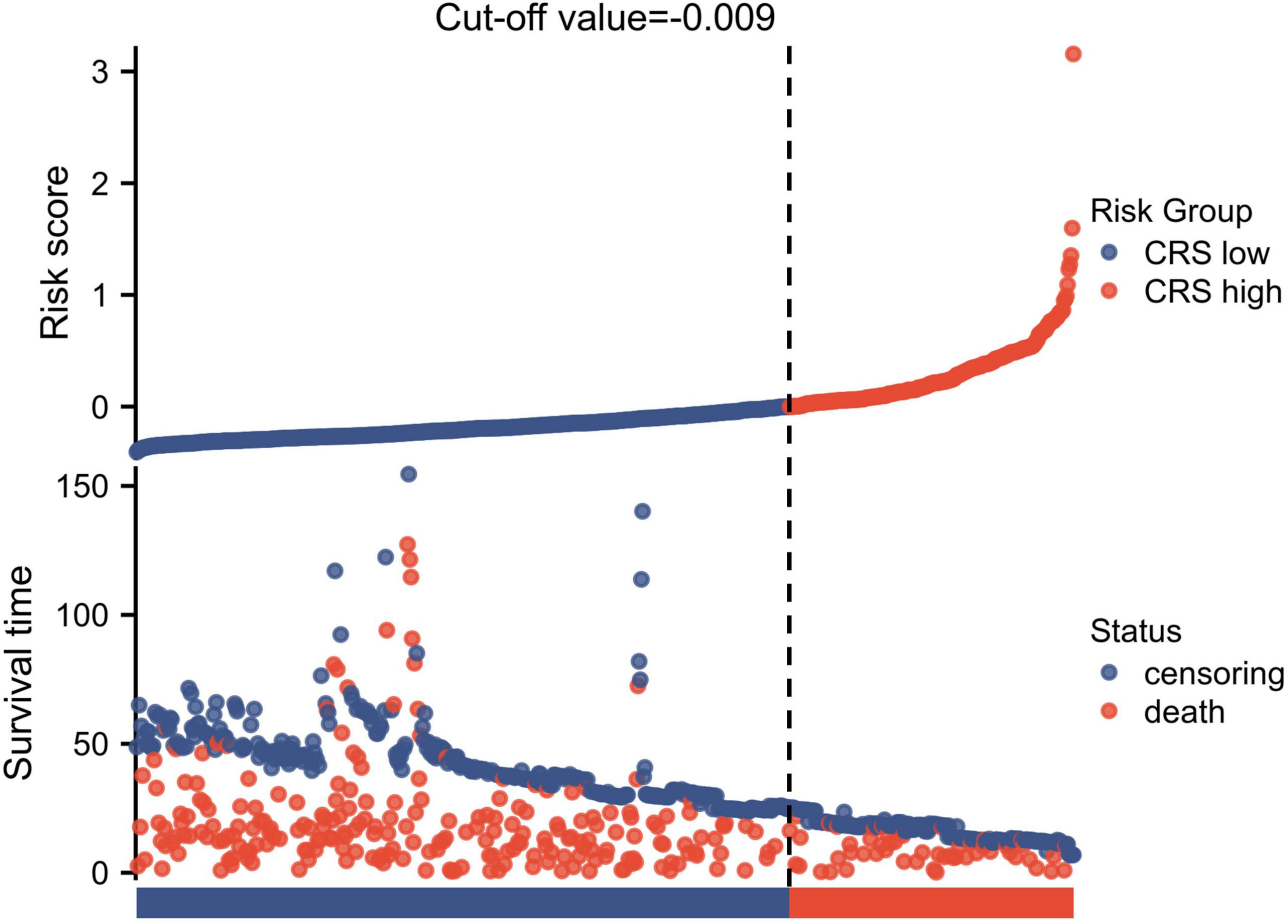
The cutoff value for CRS was determined using the Youden index applied to OS.

Following the inclusion and exclusion criteria outlined in the present study, 814 patients were included in the final analysis (**Figure 1**). The patients were categorized into two groups based on the OS cutoff CRS (-0.009) (**Figure 2**). The low and high CRS groups comprised 258 and 556 patients, respectively. Demographic and oncological characteristics of patients are presented in **Table 1**. The median CRS was -0.211 in the low CRS group and 0.181 in the high CRS group. Patients in the CRS-high group exhibited significantly older age, a higher proportion of males, elevated performance status (PS) and American Society of Anesthesiologists physiological status (ASA) scores, and severer comorbidities. In the clinicopathological aspects, the CRS-high group exhibited a higher incidence of esophageal adenocarcinoma, larger tumor sizes, increased lymphatic invasion rates, and advanced TRS staging. Additionally, we observed significant differences in surgical procedure, operative time, and intraoperative blood loss for different CRS groups. Of note, patients with elevated CRS scores demonstrated markedly heightened rates of recurrence and cancer-related mortality compared to the low CRS group.

**Table 1:** Demographic and oncological characteristics.

Characteristics	Patients Cohort, No. ( / SD)		P value
	CRS high (n=258)	CRS low (n=556)	
Age			
	63.6 (7.60)	61.8 (7.91)	0.002
Sex			
Female	28 (10.9)	104 (18.7)	
Male	230 (89.1)	452 (81.3)	0.006
BMI (kg/m2)			
	22.1 (3.30)	22.4 (3.07)	0.114
Average PS score			
	1.29 (1.06)	0.356 (0.516)	<0.001
Average ASA score			
	1.77 (0.731)	1.37 (0.521)	<0.001
Comorbidities			
Severe heart disease	27 (10.5)	1 (0.2)	<0.001
Severe pulmonary disease	20 (7.8)	2 (0.4)	<0.001
Diabetes mellitus	41 (15.9)	23 (4.1)	<0.001
Operation time (minutes)			
	286 (89.8)	259 (56.0)	<0.001
Bleeding (ml)			
	148 (304)	66.4 (44.5)	<0.001
Surgical procedure			
thoracoscopic	123 (47.7)	550 (98.9)	
thoracotomy	96 (37.2)	6 (1.1)	
hybrid	39 (15.1)	0 (0)	<0.001
Complication			
None or CD grade I	191 (74.0)	532 (95.7)	
CD grade II or higher	67 (26.0)	24 (4.3)	<0.001
Primary site			
Upper	34 (13.2)	62 (11.2)	
Middle	128 (49.6)	315 (56.7)	
Lower	96 (37.2)	179 (32.2)	0.171
Histologic type			
Adenocarcinoma	50 (19.4)	17 (3.1)	
SCC	201 (77.9)	520 (93.5)	
Others	7 (2.7)	19 (3.4)	<0.001
pT stage	393 (91.0)	196 (90.7)	
T0	58 (22.5)	202 (36.3)	
T1	35 (13.6)	84 (15.1)	
T2	39 (15.1)	80 (14.4)	
T3	126 (48.8)	190 (34.2)	<0.001
pN stage	393 (91.0)	196 (90.7)	
N0	149 (57.8)	349 (62.8)	
N1	69 (26.7)	127 (22.8)	
N2	25 (9.7)	64 (11.5)	
N3	15 (5.8)	16 (2.9)	0.095
TNM stage			
I-II	149 (57.8)	349 (62.8)	
III-IV	109 (42.2)	207 (37.2)	0.197
TRS			
TRS0-1	101 (39.1)	295 (53.1)	
TRS2-3	157 (60.9)	261 (46.9)	<0.001
Lymphatic invasion			
No	119 (46.1)	351 (63.1)	
Yes	139 (53.9)	205 (36.9)	<0.001
Recurrence			
No	128 (49.6)	361 (64.9)	
Yes	130 (50.4)	195 (35.1)	<0.001
Cancer-related death			
No	180 (69.8)	434 (78.1)	
Yes	78 (30.2)	122 (21.9)	0.013

Categoric data are shown as number (%) and continuous data as mean ± standard deviation; SD, standard deviation; CRS, comprehensive risk score; BMI, body mass index; PS, performance status; ASA, American Society of Anesthesiologists physiological status; CD, Clavien–Dindo; TRS, tumor regression scores.

### E− PASS and postoperative complications

**Figure 3:**
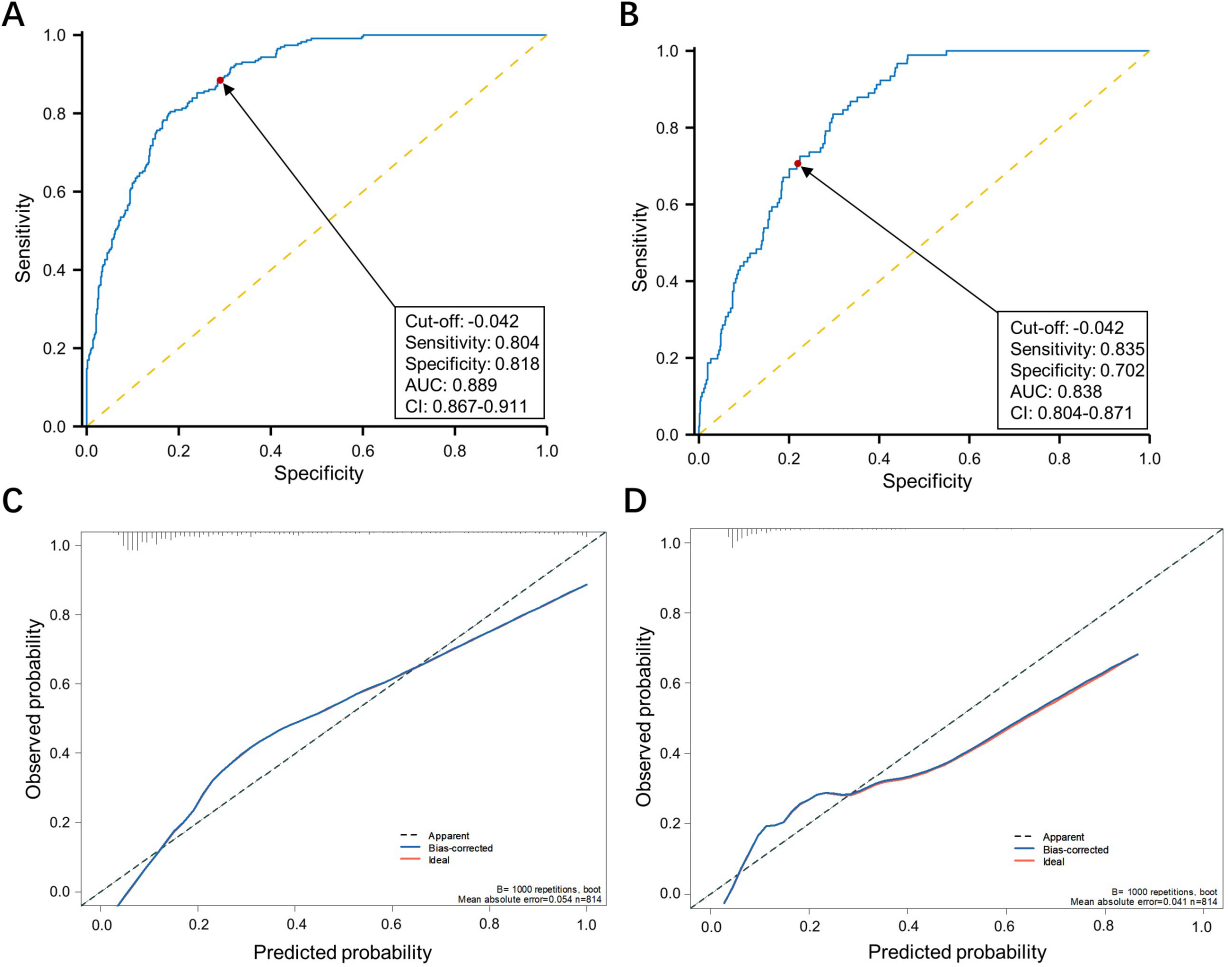
Assessing the efficacy of the E-PASS model in predicting postoperative complications. Receiver operating characteristic curve of the E-PASS as a predictive factor of overall and ≥ Clavien-Dindo II postoperative complications **(A, B)**. The calibration curve depicts the agreement between the predicted and observed outcomes. B = 1000 repetitions, boot mean absolute error = 0.054 and 0.041 respectively, n = 814 **(C, D)**.

**Figure 4:**
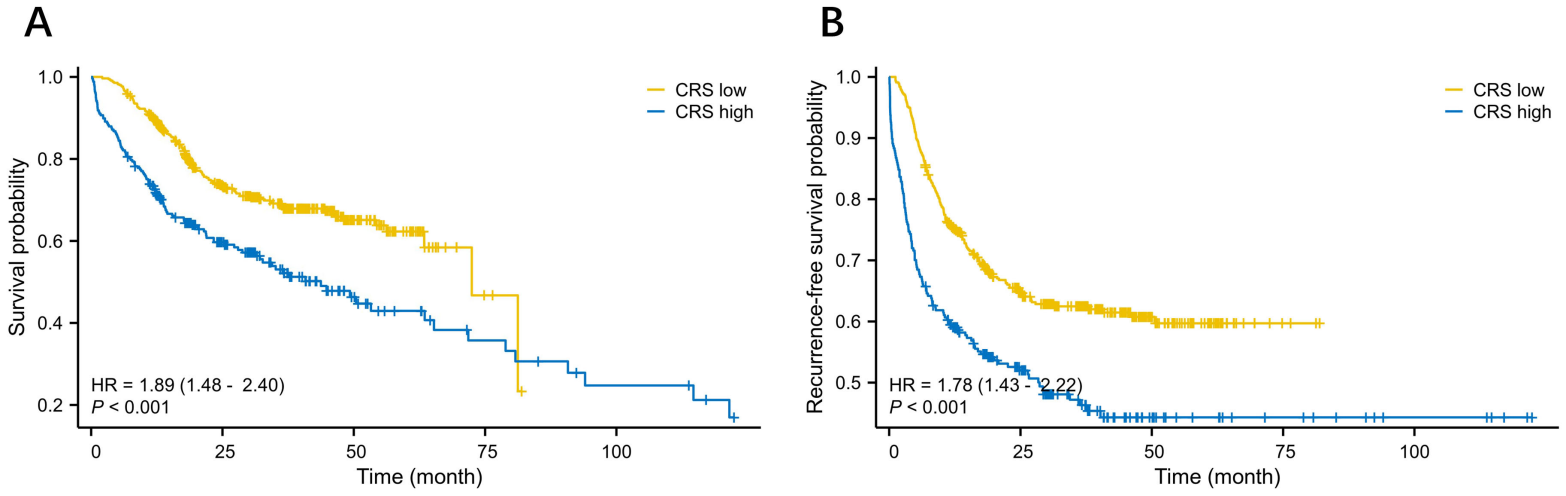
The clinical utility of the ROC curves. the DCA analysis illustrated the potential of the E-PASS model in predicting the actual clinical efficiency for overall and ≥ Clavien-Dindo II postoperative complications **(A)** and **(C)**. the CIC analysis illustrated the potential of the E-PASS model in predicting the patient benefit rates overall and ≥ Clavien-Dindo II postoperative complications **(B)** and **(D)**.

We also observed that the incidence of grade II or more postoperative complications in the CRS high group were significantly higher than the CRS-low group (26.0% vs 4.3%, p<0.001) (**Table 1**). Noting this, we utilized the E-PASS model to predict postoperative morbidity in patients underwent nCRT combined with curative esophagectomy. ROC curves were employed to evaluate the discriminative power. Areas under the curve for overall and severe postoperative morbidity were 0.889 (95% CI, 0.867-0.911; cutoff value, -0.042; sensitivity, 80.4%; specificity, 81.8%) and 0.838 (95% CI, 0.804-0.87; cutoff value, -0.042; sensitivity, 83.5%; specificity, 70.2%), respectively, indicating acceptable discrimination (**Figures 3A, B**). The calibration plot demonstrated that the predicted probabilities of the ROC aligned well with the ideal state, signifying good calibration of the model (**Figures 3C, D**). Furthermore, the DCA and CIC analysis illustrated the potential of the E-PASS model in predicting the actual clinical efficiency and patient benefit rates for postoperative morbidity (**Figure 4**).

### E− PASS and long-term outcomes

We analyzed the relationship between OS as well as RFS and clinicopathological factors with the Cox regression model. In the univariate analysis, factors such as gender, ≥ CD II postoperative complications, T2 and T3 tumor invasion, any stage of lymph node (LN) metastasis, high TRS and CRS subgroup were significantly associated with OS. In the multivariate analysis, ≥ CD II postoperative complications, LN metastasis, and high CRS emerged as independent prognostic factors for OS (**Table 2**). Subsequently, we investigated the association between RFS and relevant factors using the Cox regression model. In the univariate analysis, gender, postoperative complications of CD grade II or higher, T2 and T3 tumor invasion, LN metastasis, high TRS and high CRS subgroup were significantly associated with OS. The multivariate analysis confirmed that postoperative complications of CD grade II or higher, LN metastasis, and high CRS remained independent prognostic factors for OS (**Table 3**). Our analysis indicated that LN metastasis, severe postoperative complications, and high CRS were robust predictors significantly associated with poor long-term outcomes. **Figure 4** depict the Kaplan–Meier survival curves of OS and RFS between the low and high CRS groups. For OS, the survival curve of patients according to the CRS was significantly different between the groups (HR 1.89, 95% CI: 1.48 - 2.40; P <0.001) (**Figure 4A**). Additionally, as shown in **Figure 4B**, patients with higher CRS were significantly associated with worse RFS (HR 1.78, 95% CI: 1.43 - 2.22; P <0.001) in our cohort. Additionally, we evaluated the prognostic value of CRS when treated as a continuous variable. The results demonstrated that CRS, as a continuous metric, was also predictive of long-term postoperative outcomes (**Supplementary Tables 1, 2**).

**Table 2:** Univariate and multivariate analysis for overall survival.

Characteristics	Univariate		Multivariate	
	HR (95% CI)	P value	HR (95% CI)	P value
Gender				
Male	Reference		Reference	
Female	1.889 (1.293 - 2.760)	0.001	1.235 (0.840 - 1.818)	0.284
Age	1.003 (0.987 - 1.018)	0.741		
Complications				
None or CD grade I	Reference		Reference	
CD grade II or higher	2.109 (1.548 - 2.871)	<0.001	2.076 (1.496 - 2.883)	<0.001
pT stage				
T0	Reference		Reference	
T1	0.998 (0.611 - 1.632)	0.995	0.703 (0.418 - 1.184)	0.186
T2	1.669 (1.095 - 2.545)	0.017	1.205 (0.706 - 2.058)	0.494
T3	3.136 (2.294 - 4.287)	<0.001	1.518 (0.910 - 2.533)	0.11
pN stage				
N0	Reference		Reference	
N1	2.792 (2.115 - 3.687)	<0.001	2.514 (1.884 - 3.354)	<0.001
N2	4.215 (3.042 - 5.841)	<0.001	3.637 (2.554 - 5.181)	<0.001
N3	8.709 (5.545 - 13.677)	<0.001	6.423 (4.005 - 10.300)	<0.001
TRS				
TRS0-1	Reference		Reference	
TRS2-3	2.573 (1.992 - 3.324)	<0.001	1.170 (0.761 - 1.799)	0.474
CRS grade				
Low	Reference		Reference	
High	1.886 (1.483 - 2.398)	<0.001	1.486 (1.148 - 1.925)	0.003

HR, Hazard ratio; CI, Confidence interval; CRS, comprehensive risk score; CD, Clavien–Dindo; TRS, tumor regression scores.

**Table 3:** Univariate and multivariate analysis for recurrence-free survival.

Characteristics	Univariate		Multivariate	
	HR (95% CI)	P value	HR (95% CI)	P value
Gender			Reference	
Male	Reference			
Female	1.554 (1.116 - 2.164)	0.009	1.060 (0.756 - 1.488)	0.735
Age	0.993 (0.979 - 1.008)	0.361		
Complications				
None or CD grade I	Reference		Reference	
CD grade II or higher	2.110 (1.572 - 2.832)	<0.001	2.022 (1.481 - 2.761)	<0.001
pT stage				
T0	Reference		Reference	
T1	1.014 (0.657 - 1.565)	0.949	0.715 (0.449 - 1.137)	0.156
T2	1.882 (1.300 - 2.724)	<0.001	1.290 (0.804 - 2.069)	0.291
T3	2.988 (2.245 - 3.975)	<0.001	1.507 (0.946 - 2.402)	0.084
pN stage				
N0	Reference		Reference	
N1	2.609 (2.022 - 3.367)	<0.001	2.334 (1.794 - 3.038)	<0.001
N2	3.533 (2.595 - 4.811)	<0.001	3.016 (2.165 - 4.201)	<0.001
N3	6.635 (4.340 - 10.143)	<0.001	4.792 (3.083 - 7.449)	<0.001
TRS				
TRS0-1	Reference		Reference	
TRS2-3	2.488 (1.969 - 3.145)	<0.001	1.246 (0.846 - 1.836)	0.266
CRS grade				
Low	Reference		Reference	
High	1.781 (1.426 - 2.224)	<0.001	1.443 (1.139 - 1.829)	0.002

HR, Hazard ratio; CI, Confidence interval; CRS, comprehensive risk score; CD, Clavien–Dindo; TRS, tumor regression scores.

To investigate whether the predominance of operative versus physical burden within the same CRS level influences long-term survival outcomes, we stratified patients based on their PRS (Physical Risk Score) and SSS (Surgical Severity Score) within each CRS (Combined Risk Score) category. Long-term survival analyses were then performed across subgroups. Among patients with high CRS levels, no significant difference in long-term survival was observed between those with predominant surgical burden and those with predominant physical burden. However, in patients with low CRS levels, a higher SSS appeared to be associated with a trend toward poorer long-term prognosis (**Supplementary Figure 1**).

Finally, the association between CRS and patients prognosis with different TRS and TNM stage was examined through Kaplan–Meier analysis. The results indicated that elevated CRS was consistently linked to poorer OS and RFS across both early and advanced TNM and TRS stage patients (**Supplementary Figures 2, 3**).

## Discussion

Previous research on the relationship between esophageal cancer and the E-PASS model has primarily focused on the association with esophagectomy and in-hospital and postoperative morbidity (17, 19). This study represents the first investigation focusing on patients with nCRT and curative esophagectomy, which now gradually become the standard treatment for advanced esophageal cancer patients, exploring the relationship between the E-PASS model and short-term and long-term outcomes. Our investigation indicated that the E-PASS scoring system can serve as a visible tool for preoperative risk assessment and shared decision-making. Additionally, it underscored the significance for thoracic surgeons to comprehensively assess surgical risks based on each patient’s physical state to make informed decisions regarding suitable surgical opportunities.

In the past decades, advancements in esophageal cancer curative surgery, combined with progress in minimally invasive techniques and postoperative care management, have reduced surgical risks in subgroups of patients (20, 21). Furthermore, nCRT has significantly benefit survival rates following esophagectomy (22). Notwithstanding these advancements, the 5-year OS after esophagectomy remain suboptimal, ranging from 34% to 48.2% in high-volume centers (23–25). Notably, patients receiving nCRT may face higher surgical risks, potentially leading to fatal complications like esophageal perforation or bleeding. Such surgeries with potential risk factors necessitate thorough patient counseling and assessment, as these elevated morbidity and mortality rates may be deemed unacceptable for certain patients.

To date, there hasn’t been a dedicated operative risk model specifically tailored for esophageal cancer. The E-PASS model was initially developed across a broad spectrum of gastrointestinal surgical procedures and has demonstrated superior predictive performance compared to the well-known Physiological and Operative Severity Score for the Enumeration of Mortality and Morbidity (POSSUM) in terms of mortality and morbidity rates (13, 15, 16). Previous research has also shown that the E-PASS model is more effective in predicting severe morbidity in elderly patients (aged ≥70) compared to the modified E-PASS (mE-PASS) and POSSUM models (26). Therefore, for esophageal cancer patients with a peak incidence of morbidity between 70–79 years, we utilized the E-PASS model (27, 28). Yamashita et al. and Yoshida et al. have previously documented the significant value of E-PASS in predicting postoperative complication in esophageal cancer (17, 29). However, these studies have limitations due to the heterogeneity of the populations and small sample sizes, and at that time, nCRT was not a standard treatment for esophageal cancer. Therefore, this study, which includes 814 patients from a well-defined nCRT cohort, adds substantial weight to the value of the E-PASS models in predicting postoperative morbidity and mortality in esophageal cancer.

Despite the lack of esophageal cancer-specific factors in the E-PASS model, CRS demonstrated acceptable discriminative ability for postoperative morbidity in our cohort. In previous investigations, the major focus in E-PASS model related research has been on its association with postoperative morbidity (30, 31). CRS is commonly acknowledged as a reliable predictor for postoperative complications, which aligning with our findings based on overall and severe postoperative complication. Of note, we also validated the potential of the E-PASS model in predicting the actual clinical efficiency and patient benefit rates for postoperative morbidity. Compared to POSSUM, E-PASS generally demonstrated superior predictive capabilities for short-term outcomes (32, 33). E-PASS incorporates more objective cardiopulmonary functional indicators, adjusts intraoperative blood loss for patient weight, and includes continuous variables in physiological and surgical factors. These strengths might contribute to EPASS outperforming POSSUM in predicting short-term outcomes, but further research is needed to comprehensively compare the effectiveness of these scoring systems.

Regarding long-term outcomes, we observed that E-PASS predicts long-term survival in patients undergoing esophagectomy after nCRT, irrespective of esophageal cancer TNM and TRS stages. The E-PASS score, known as CRS, comprises the PRS and SSS. The PRS includes age, severe heart disease, severe pulmonary disease, diabetes, performance status, and the ASA classification. Therefore, it is reasonable to consider that these baseline factors are associated with patients postoperative OS. Furthermore, our findings indicated that the CRS is related to the RFS time. This association can be explained by the fact that patients with advanced esophageal cancer often have higher SSS, as surgery requires longer operative times, and advanced-stage patients tend to experience greater intraoperative blood loss. Additionally, minimally invasive approaches are more commonly employed in the treatment of early-stage esophageal cancer. Despite the longer operative time associated with this approach, most cases are classified into the CRS low group due to reduced intraoperative blood loss and smaller skin incisions. To address these confounding factors, we performed subgroup and multivariate analyses. Our results demonstrate that E-PASS is an independent prognostic factor across the entire cancer stages. This indicated that the association between E-PASS and prognosis extends beyond advanced esophageal cancer.

However, the E-PASS model has inherent limitations that warrant consideration for potential optimization. Firstly, given its focus on multiple surgical variables, the model may be suboptimal for preoperative risk assessment. Estimating these variables preoperatively might lead to underestimation or overestimation of surgical outcomes. Additionally, key factors crucial for esophageal cancer prognosis, such as anastomotic reconstruction and anastomotic conditions, are not included, potentially affecting the model’s predictive accuracy. Lastly, while E-PASS demonstrates efficacy in predicting postoperative complications in minimally invasive procedures, its capability to comprehensively evaluate long-term survival outcomes may be constrained due to the lower prevalence of minimally invasive procedures in patients with advanced esophageal cancer. Nevertheless, recognizing this limitation provides valuable insights for the prospective development of specialized prognostic models tailored specifically for curative esophageal cancer surgery.

This study has several limitations that warrant discussion. Firstly, the retrospective and observational nature of this study introduces inherent flaws in data analysis. Secondly, the use of the Youden index applied to the OS curve to obtain the CRS cutoff value has not been validated internally and externally. Thirdly, the distinct heterogeneity present in esophageal tumors of different pathological types underscores the need for comprehensive research on each subtype to validate the generalizability of the prediction model. Finally, E-PASS model is that certain operative variables used to calculate the SSS—such as blood loss, operative time, and incision type—are only available postoperatively, limiting the model’s direct applicability in preoperative decision-making. While our study has certain drawbacks, it may still serve as a valuable conclusion for future research. Based on our findings, we plan to develop a preoperative and intraoperative risk model for postoperative morbidity and long-term outcome following esophageal cancer resection.

In conclusion, the current investigation suggested that the E-PASS model can accurately identify patients following nCRT combined with curative esophagectomy, who are prone to higher postoperative complication rates and exhibit inferior long-term prognosis. In the absence of models specific to esophageal cancer and its associated risk factors, the EPASS model facilitates risk assessment and patient counseling during the initial outpatient visit, thereby supporting shared decision-making.

## Data Availability

The raw data supporting the conclusions of this article will be made available by the authors, without undue reservation.
