# Supplementary material for: Comprehensive risk score of the E-PASS scoring system serves a prognostic indicator for patients after neoadjuvant therapy and curative esophageal cancer surgery: a multicenter retrospective study
Source: Front Oncol. 2025 Jun 6;15:1617683. doi: 10.3389/fonc.2025.1617683 (PMC12203562; doi:10.3389/fonc.2025.1617683)
Supplement: Supplementary file 1 [file DataSheet1.docx]

**Supplementary Table.**

**Supplementary table1.** Univariate and multivariate analysis for overall survival, CRS as continuous value.

|  | Univariate | | Multivariate | |
| --- | --- | --- | --- | --- |
| Characteristics | HR (95% CI) | P value | HR (95% CI) | P value |
| Gender |  |  |  |  |
| Male | Reference |  | Reference |  |
| Female | 1.889 (1.293 - 2.760) | 0.001 | 1.332 (0.903 - 1.964) | 0.148 |
| Age | 1.003 (0.987 - 1.018) | 0.741 |  |  |
| Complication |  |  |  |  |
| None or CD grade I | Reference |  | Reference |  |
| CD grade II or higher | 2.109 (1.548 - 2.871) | < 0.001 | 2.366 (1.724 - 3.246) | < 0.001 |
| pT stage |  |  |  |  |
| T0 | Reference |  | Reference |  |
| T2 | 1.669 (1.095 - 2.545) | 0.017 | 1.156 (0.676 - 1.974) | 0.596 |
| T3 | 3.136 (2.294 - 4.287) | < 0.001 | 1.550 (0.929 - 2.586) | 0.093 |
| T1 | 0.998 (0.611 - 1.632) | 0.995 | 0.687 (0.407 - 1.160) | 0.16 |
| pN stage |  |  |  |  |
| N0 | Reference |  | Reference |  |
| N2 | 4.215 (3.042 - 5.841) | < 0.001 | 3.428 (2.414 - 4.867) | < 0.001 |
| N1 | 2.792 (2.115 - 3.687) | < 0.001 | 2.507 (1.880 - 3.344) | < 0.001 |
| N3 | 8.709 (5.545 - 13.677) | < 0.001 | 5.996 (3.721 - 9.661) | < 0.001 |
| TRS |  |  |  |  |
| TRS0-1 | Reference |  | Reference |  |
| TRS2-3 | 2.573 (1.992 - 3.324) | < 0.001 | 1.231 (0.804 - 1.884) | 0.339 |
| CRS value | 1.429 (1.116 - 1.829) | 0.005 | 1.250 (0.969 - 1.611) | 0.086 |

HR, Hazard ratio; CI, Confidence interval; CRS, comprehensive risk score; CD, Clavien–Dindo; TRS, tumor regression scores.

**Supplementary table2.** Univariate and multivariate analysis for recurrence-free survival, CRS as continuous value.

|  | Univariate | | Multivariate | |
| --- | --- | --- | --- | --- |
| Characteristics | HR (95% CI) | P value | HR (95% CI) | P value |
| Gender |  |  |  |  |
| Male | Reference |  | Reference |  |
| Female | 1.554 (1.116 - 2.164) | 0.009 | 1.140 (0.812 - 1.599) | 0.45 |
| Age | 0.993 (0.979 - 1.008) | 0.366 |  |  |
| Complication |  |  |  |  |
| None or CD grade I | Reference |  | Reference |  |
| CD grade II or higher | 2.110 (1.572 - 2.832) | < 0.001 | 2.270 (1.680 - 3.068) | < 0.001 |
| pT stage |  |  |  |  |
| T0 | Reference |  | Reference |  |
| T2 | 1.891 (1.306 - 2.738) | < 0.001 | 1.265 (0.788 - 2.030) | 0.33 |
| T3 | 2.992 (2.249 - 3.981) | < 0.001 | 1.541 (0.968 - 2.452) | 0.068 |
| T1 | 1.016 (0.658 - 1.567) | 0.944 | 0.707 (0.443 - 1.127) | 0.145 |
| pN stage |  |  |  |  |
| N0 | Reference |  | Reference |  |
| N2 | 3.537 (2.597 - 4.815) | < 0.001 | 2.853 (2.055 - 3.962) | < 0.001 |
| N1 | 2.618 (2.029 - 3.379) | < 0.001 | 2.312 (1.777 - 3.008) | < 0.001 |
| N3 | 6.642 (4.344 - 10.155) | < 0.001 | 4.437 (2.844 - 6.922) | < 0.001 |
| TRS |  |  |  |  |
| TRS0-1 | Reference |  | Reference |  |
| TRS2-3 | 2.494 (1.973 - 3.152) | < 0.001 | 1.298 (0.884 - 1.906) | 0.183 |
| CRS value | 1.474 (1.169 - 1.860) | < 0.001 | 1.257 (0.989 - 1.597) | 0.078 |

HR, Hazard ratio; CI, Confidence interval; CRS, comprehensive risk score; CD, Clavien–Dindo; TRS, tumor regression scores.

**Supplementary Figure.**

**
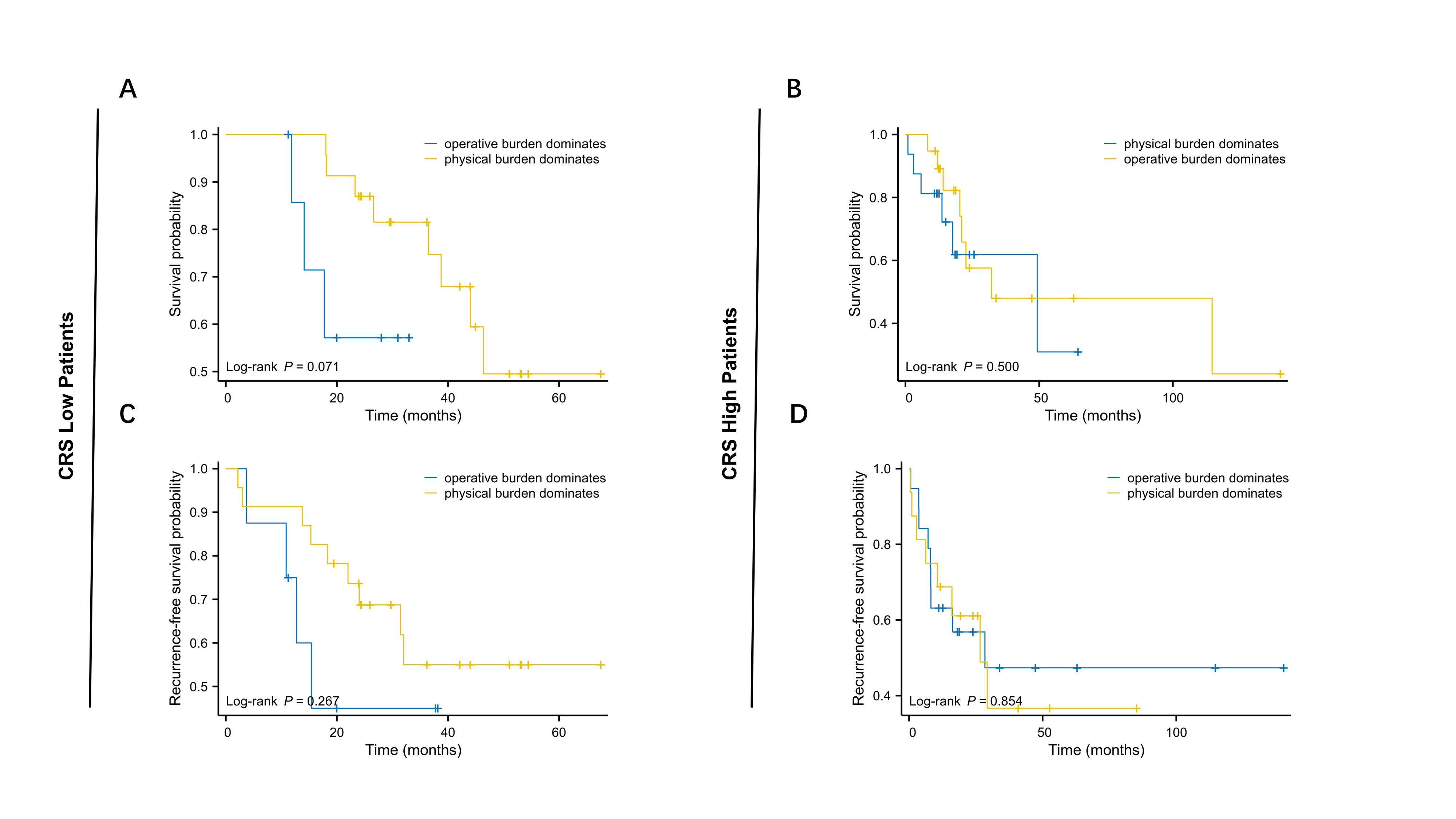
**

**Supplementary Fig.1** Kaplan‒Meier survival curve for OS and RFS in different CRS patients stratified by predominance of operative versus physical burden.


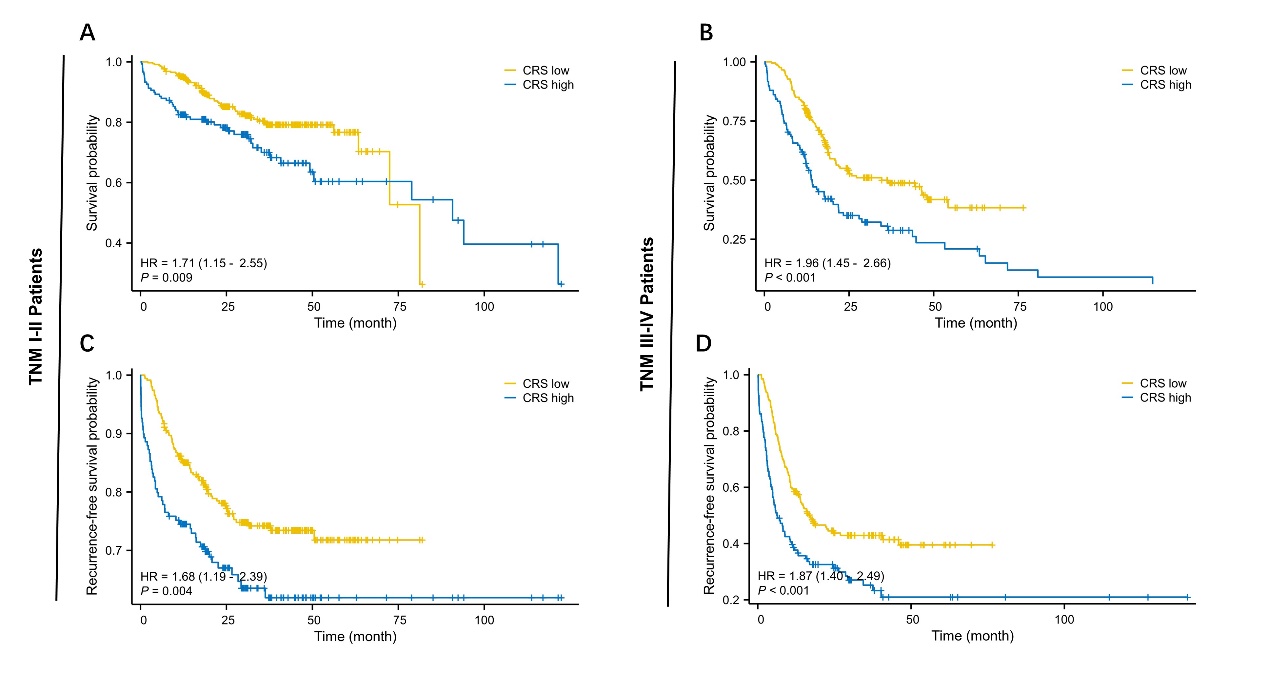


**Supplementary Fig.2** Kaplan‒Meier survival curve for OS and RFS in different TNM stages patients stratified by CRS.


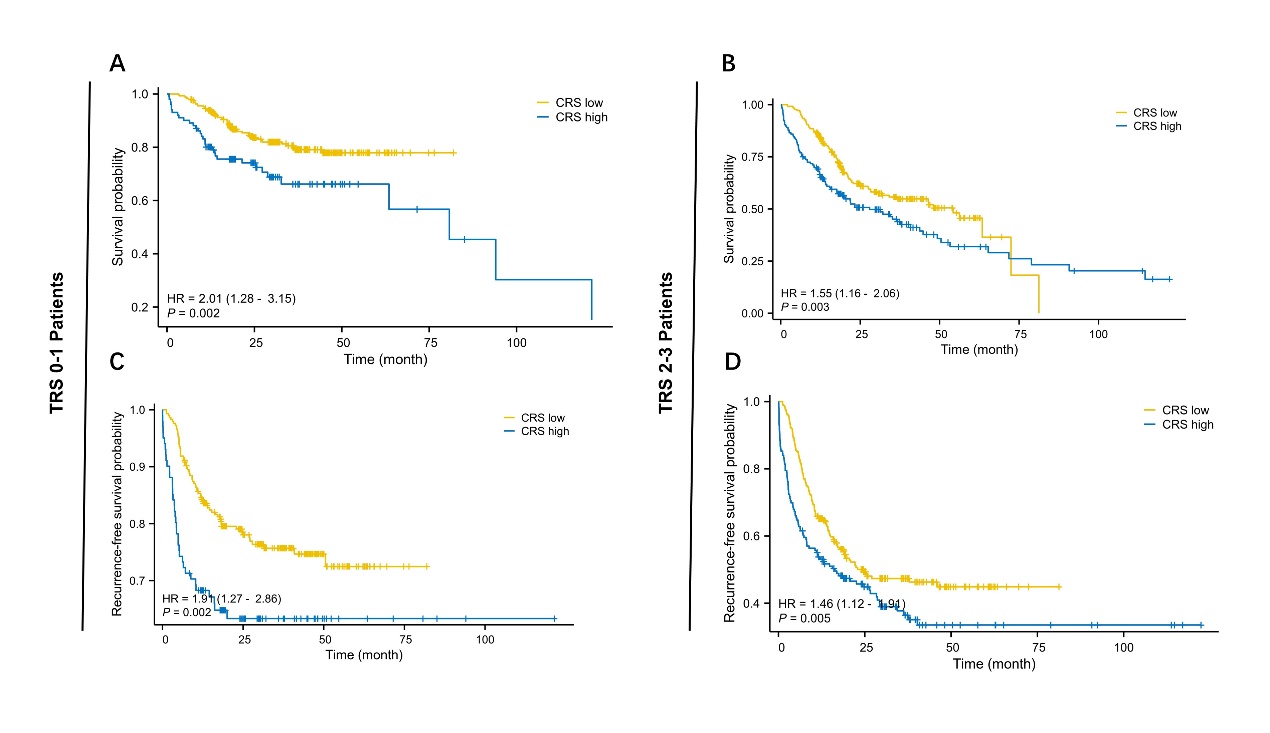


**Supplementary Fig.3** Kaplan‒Meier survival curve for OS and RFS in different TRS stages patients stratified by CRS.
